# A randomized comparison of the prone ventilation endotracheal tube versus the traditional endotracheal tube in adult patients undergoing prone position surgery

**DOI:** 10.1038/s41598-017-02006-6

**Published:** 2017-05-11

**Authors:** Wangyuan Zou, Jiali Shao, Xia Liang, Lin Li, Zhenghua He, Qulian Guo

**Affiliations:** Department of Anesthesiology, Xiangya Hospital, Central South University, Changsha, Hunan 410008 China

## Abstract

Endotracheal tube displacement or dislocation is a severe complication that can occur in patients who require prone position ventilation. We hypothesized the prone position tube (PPT) would reduce the incidence of displacement of an endotracheal tube in an adult prone operation compared to a traditional tube (TT). A total of 80 adult patients undergoing neurosurgery or spine surgery were recruited. Sixty patients with prone position ventilation were randomly divided into the traditional routine endotracheal tube group (Group TT, n = 30) and the prone position ventilation endotracheal tube group (Group PPT, n = 30). The primary outcome measures were the incidence of the endotracheal tube displacement during surgery, and the secondary outcomes were symptoms of sore throat, dysphagia and dysphonia during follow-up in the post-anesthesia care unit (PACU). The incidence of tube displacement was significantly lower in the PPT group (0 [0%] of 30 patients) compared to the TT group (22 [73.3%] of 30 patients; odds ratio [OR] 0.73, 95% CI 0.591–0.910; *P* = 0.005). There was no statistical difference in sore throat, dysphagia and vocal function between the two groups (*P* > 0.05) during follow-up. Compared to the traditional tube, the improved prone positon tube reduced the incidence of displacement of the endotracheal tube. This study was registered with ClinicalTrials.gov on April 29, 2015 (No. NCT02449356**)**.

## Introduction

Endotracheal tube displacement is one of the leading causes for airway-related complications. Conventional endotracheal intubation in patients undergoing prone position surgery has the risk of displacement or dislocation of the endotracheal tube. The related causes are poor fixation of endotracheal intubation, bucking and waking the patients due to light anesthesia during surgery. In this situation, re-fixation or reintubation may be difficult because of the prone position^1^. Therefore, the instability of the endotracheal tube in patients in the prone position is potentially life threatening^2, 3^. Anesthesiologists should focus on managing major intraoperative emergencies (e.g., accidental extubation) and anticipate postoperative complications^4^.

In prone position surgery (usually neurosurgery or spinal surgery), a traditional endotracheal tube is usually used, and then medical adhesive tapes are used to fix the tube wrapping around the bite-block and stick it on the skin around the mouth, or by a cord binding the tube to the bite-block. During surgery in the prone position, the tube may be displaced because of the gravity effect, and the tape may become damp from oral secretion, so the tube cannot be secured with this method and may result in severe displacement or dislocation of the tube as well as catastrophic airway complications.

**Figure 1:**
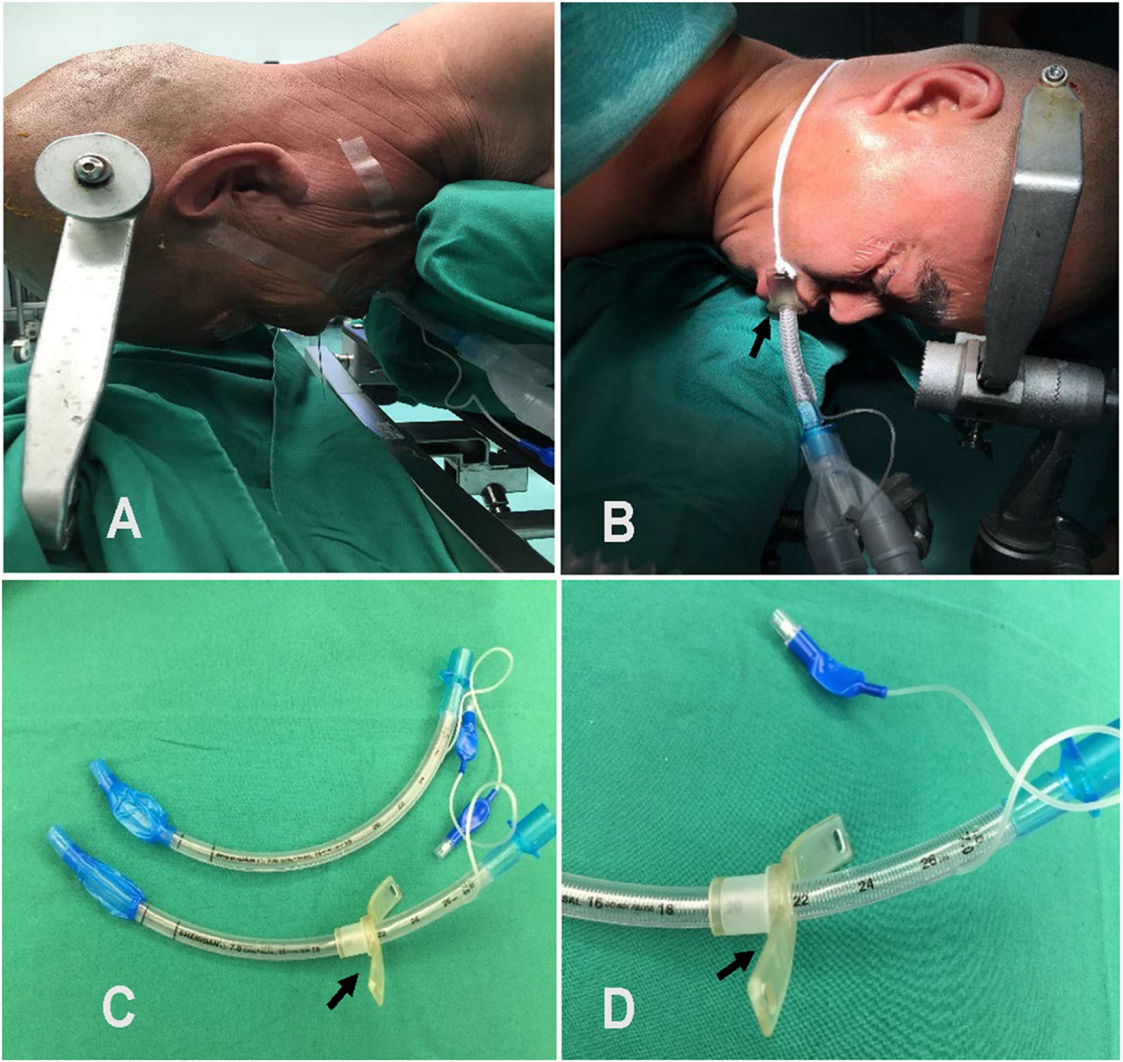
Photograph showing the traditional tube used in an operation (**A**) and the prone position tube used in an operation (**B**). The prone position tube (PPT) and the traditional tube (TT) (**C**). With a highlighted image of the prone position tube (PPT) (**D**).

Therefore, determining how to prevent the displacement or dislocation of the tube and ensure the airway patency of the patient should be emphasized. It is urgent to find a way to ensure the security of the prone ventilation. In our study, we applied a custom-designed prone position tube (PPT), which was different from the conventional endotracheal tube, including two parts of the fixed device and the traditional endotracheal tube. The fixed device had one hole on each side, and the connecting cord was tied to fix the PPT (Fig. 1).

In our previous research, we conducted a trial in pediatric patients for prone position ventilation, and the results indicated that the prone position tube (PPT) could reduce the incidence of airway complications compared to a traditional tube (TT)^5^. In this study, we compared PPT to TT in adult patients undergoing an operation in the prone position to determine whether the PPT had the same merits in adult patients and whether it decreased the incidence of serious peri-anesthetic complications and provided effective airway protection.

## Cases and Methods

### Cases

This study was conducted after obtaining approval from the Ethics Committee of Xiangya Hospital affiliated with Central South University. Written informed consent form was obtained from all the patients prior to conducting any study procedures. The study was performed in accordance with the approved guidelines and regulations.

**Figure 2:**
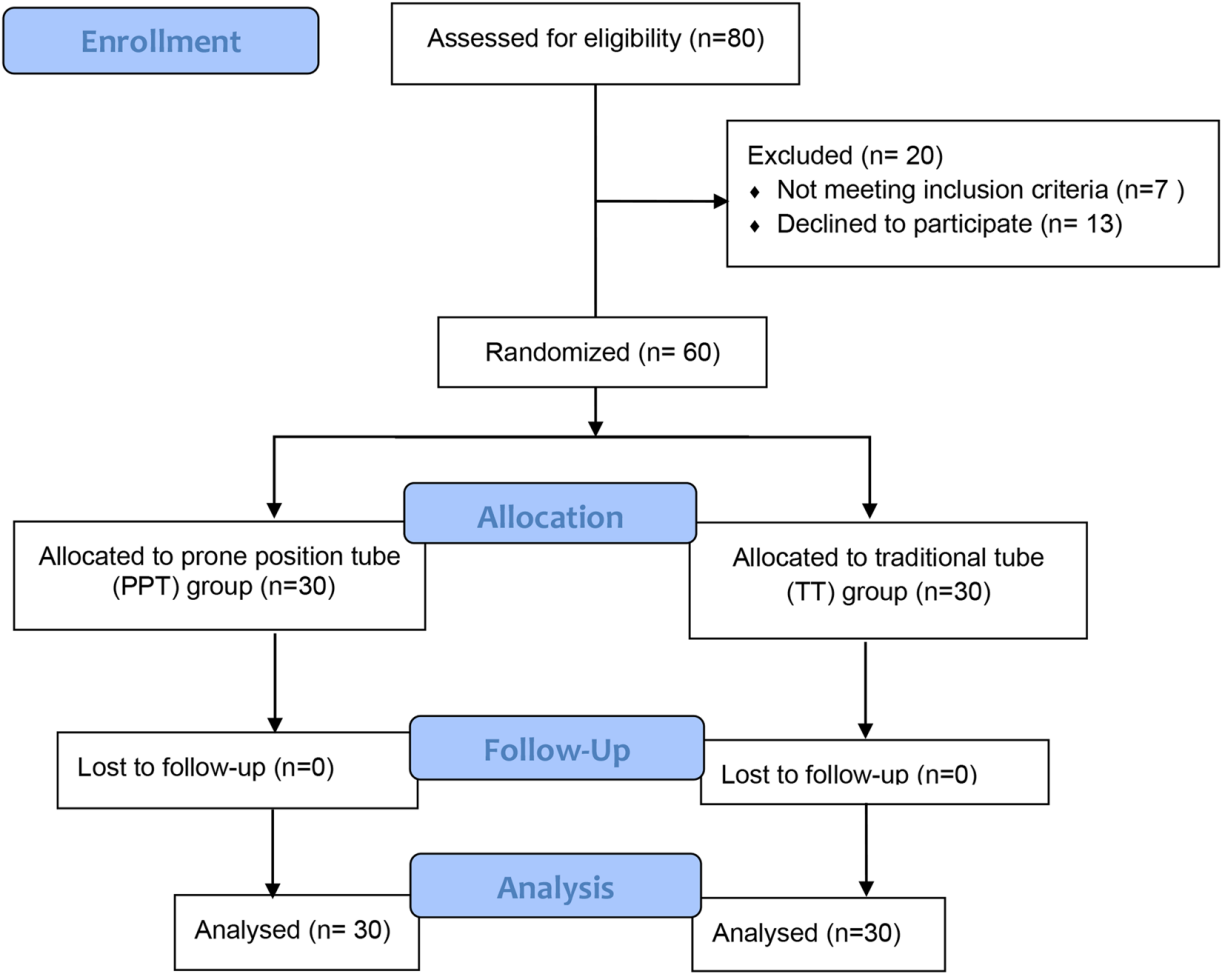
Trial flow diagram.

Sixty adult patients with ages ranging from 18–65 years old who were receiving neurosurgery or spinal surgery were recruited for the study. Their weights ranged from 50 to 75 kilograms; there were 31 males and 29 females, and all were ASA grade I or II. These patients were randomly divided into two groups, including the prone position ventilation endotracheal tube group (Group PPT, n = 30) and the traditional endotracheal tube ventilation group (Group TT, n = 30) (Fig. 2). The general information for the patients is listed in Table 1. This trial was conducted from September 2015 to September 2016. The study recruited participants through advertisements. The subjects were simply randomized (through a random number table).

**Table 1:** The general information of the patients.

Variable	Prone position tube	Traditional tube	*p*-value
Age (yr)	49 ± 5	47 ± 6	>0.05
Height (cm)	171 ± 20	168 ± 23	>0.05
Weight (kg)	67 ± 12	69 ± 16	>0.05
Gender (female/male)	15/15	14/16	>0.05
ASA grade (I/II)	16/14	15/15	>0.05
Insertion time (sec)	19 ± 4	21 ± 6	>0.05
Number of insertion (1/2/3)	27/3/0	28/2/0	>0.05
Duration of ventilation (min)	93 ± 23	97 ± 18	>0.05

Values are mean ± SD or number of patients. n = 30 in PPT group, n = 30 in TT group.

### Prone Ventilation Endotracheal Tube

We used the prone ventilation endotracheal tube in one group of subjects. Male adults usually had an ID 7.5–8.0 endotracheal tube and female adults had an ID 7.0–7.5 endotracheal tube, and the depth of intubation for both genders ranged from 21–24 cm from the front tooth according to the height of the patients and was confirmed by listening to their lungs.

### Anesthesia

Each patient received 0.1 g phenobarbital sodium and 0.5 mg atropine intramuscularly as premedication 30 min before anesthesia induction. After the patient arrived in the operating room, the basic monitoring measurements included BP, HR, SPO2, PetCO_2_ and ECG, and a vein was opened.

All patients received standardized general anesthesia, for the anesthesia induction, patients received a single intravenous injection of 0.05–0.1 mg/kg of midazolam, 0.4–0.5 µg/kg of sufentanil, 0.15–0.2 mg/kg of cisatracurium and 0.3–0.4 mg/kg of etomidate. For group PPT, the improved prone ventilation tube was fixed with a connecting cord around the head, which was lined by soft gauze to protect the skin from bruising. For group TT, the traditional tube was fixed by medical adhesive tapes and the bite-block. After intubation, we set the ventilator parameters, including tidal volume (8–10 ml/kg), respiratory frequency (12–15 respiratory cycles/min), PEEP zero, and mean airway pressure (12–20 mmH_2_O). Maintenance of anesthesia was achieved by a continuous intravenous injection of 4–8 mg/kg.h propofol and 4–10 µg/kg.h of remifentanil and intermittent application of sevoflurane according to the depth of anesthesia during the operation. An intermittent bolus injection of 0.05 mg/kg of atracurium was made according to the requirements of the surgery.

### Study outcomes

The primary outcome was tube displacement and bending after the head position changed and airway resistance (peak) occurred during the operation. After intubation, we made a landmark along the central upper incisors as the zero point; then, before the patients were turned over into the supine position when the surgery ended, we observed the looseness of the tape and tube displacement or dislocation and made another landmark at that end point. We used a ruler to measure the length of the displacement of the endotracheal tube. We divided the degree of the displacement of tube into 3 types, which included mild (displacement distance <0.5 cm), moderate (0.5 cm ≤displacement distance <1.5 cm), and severe (1.5 cm ≤displacement distance).

We also observed the impact of oral secretion on the stickiness of the adhesive tapes while the patient was in prone position and tube fixation after intubation. We also recorded intubation times, the ventilation time and operation times for the two groups. Meanwhile, during the operation, we observed changes in the airway pressure and the tube fixation. The secondary outcomes were sore throat, hoarseness and dysphagia in PACU (Table 2).

**Table 2:** Incidence of intraoperative or postoperative complication in patients before leaving the post-anesthesia care unit (PACU).

Variable	Mild	Traditional n = 30	Severe	Total (%)	Mild	Prone n = 30	Severe	Total (%)	*p*-value
		Medium				Medium			
Displacement of the ET	11	8	3	22 (73.3%)	0	0	0	0 (0)	<0.01
Prolapse of the ET	0	0	0	0 (0)	0	0	0	0 (0)	>0.05
Sore throat	1	0	0	1 (3.3%)	1	0	0	1 (3.3%)	0.05
Dysphagia	0	0	0	0 (0)	0	0	0	0 (0)	>0.05
Dysphonia	0	0	0	0 (0)	0	0	0	0 (0)	>0.05

Mild: <0.5 cm; Medium: 0.5–1.5 cm; Severe: >1.5 cm, ET: Endotracheal Tube.

### Statistical analysis

The primary variables were intraoperative and postoperative complications. Based on the results of a pilot study, a power analysis indicated that 30 patients per group would be sufficient to detect the difference. The data are expressed as the mean ± SD or number of patients. Patient characteristics were compared by ANOVA as appropriate. The displacement of the tubes and post-procedure complications were compared using a Chi-square test. The SPSS software (version 19.0; IBM SPSS Inc., Chicago, IL) was used. *P* < 0.05 was considered significant.

## Results

### The general information of the subjects

In all, 80 patients were willing to participate in the trial. Seven patients were not meeting inclusion criteria and 13 patients declined to participate the study. Thus, 60 participants were recruited to participate in this trial. No patients were lost during the treatment. Sixty participants completed the trial on schedule, and each group had 30 participants (Fig. 2).

The age, the proportion of gender, the duration of the operation and anesthesia had no statistically significant differences (*P* > 0.05) between the two groups (Table 1).

### The displacement of the endotracheal tube (recorded with numerical values) during the operations

In the 30 cases for group TT, various degrees of tube displacement occurred during the operations. Severe displacement of the tube occurred 3 cases and medium displacement of the tube occurred in 8 cases in group TT. The tube become bent in one case (the oxyhemoglobin saturation went down and we readjusted the location of the tube). However, the PPT group had a special prone ventilation endotracheal tube and a modified fixation method, so displacement or dislocation of the tube did not occur in any of the 30 cases. The data showed that the incidence of tube displacement was significantly lower in the PPT group (0 [0%] of 30 patients) compared to the TT group (22 [73.3%] of 30 patients; odds ratio [OR] 0.73, 95% CI 0.591–0.910; *P* = 0.005). Additionally, the tube fixation was secured in group PTT, whereas in group TT, 7 cases showed signs of the adhesive tape used for fixation becoming damp and loose, and the difference between the groups was statistically significant (*P* = 0.010) (Table 2).

### Vocal function and throat condition after surgery

There were no statistical differences in sore throat, dysphagia and vocal function between the PPT group and TT group (*P* > 0.05) (Table 2).

## Discussion

In this study, we found that compared to the traditional tube, the prone positon tube could reduce the incidence of tube displacement. The prone ventilation endotracheal tube in our study had several advantages as follows. (1) It was designed with a fixation device that is affixed to the tube to provide stabilization. (2) The fixation device and endotracheal tube was a whole unit, and the binding cord will not be affected by the sterilizing fluid or fluids leaking from the mouth or blood, which affect the tape used in the traditional method. (3) The design of the tube and fixation guarantee safe ventilation and simultaneously do not interfere with the surgical procedure, and this approach will also benefit those who are allergic to medical tape.

In this study, we found that 22 cases were displaced in group TT, and the causes were as follows. (1) Due to the special position in prone surgery, the gravity produced an outer force that played an important role in the displacement of the tube. In the prone ventilation tube, the fixation cord generated an upward force that can counteract the gravity effect acting on the tube. (2) The adhesive tape can be easily dampened by the sterilized fluid, blood, oil stains or sweat on the patient’s face. In the prone position, the oral secretion of the patients is increased and can lead to decreased adhesion between the tube, the tape and the skin. However, the fixation cord of the prone position tube remains firm, even under the damp conditions. (3) It is difficult to observe the status of the endotracheal tube while the patient is in a prone position. (4) During a prone position operation, the surgeons moving the patient’s head or sudden light anesthesia could lead to displacement of the tube. Accordingly, the fixation cord of PPT can counteract the gravity effect caused by position and decrease or avoid the incidence of risky situations for the patient^6^.

Several fixation devices of prone position ventilation have been described. A previous study reported that the endotracheal tube was secured either with adhesive tape or a Thomas tube holder, and displacement of the endotracheal tube was significantly larger in the group with adhesive tape than in the group with a Thomas holder^7, 8^. However, compared to the fixation method described above, our fixation method was more simple, more effective and easier to manage during an operation. Buckley *et al*. reported that the Haider Tube-Guard significantly reduced tube movement (>1 cm) of the endotracheal tube compared to adhesive tape and the guard was well-tolerated^9^. In our study, the displacement rate of the endotracheal tube was lower compared to the results of Buckley *et al*. This difference may have occurred because now the anesthesiologist pays more attention to the prone ventilation patients than before. Additionally, there are several points that we should take care of when using the modified fixation tube method: (1) the cord’s connecting part where the tube should be fastened; (2) continuous friction between the cord and the neck could cause rubefaction and even skin lesions, so we should put one soft gauze under the cord to protect the skin from compression^5^.

This new design of the practical prone ventilation endotracheal tube has its own matched fixtures with the tube, with locations based on the patient’s gender and age, and those features should allow the tube to match the patient. The modified prone ventilation tube can greatly reduce the risk of displacement of the tube, enhance airway safety during operations, and even protect the patient’s life. Although no bite-block was placed in the mouth, we can also conduct sputum aspiration through the space in the corners of the mouth. The endotracheal tubes in our study were steel-wire-reinforced. For those who were allergic to adhesive tape, the PPT may be a better choice. In this study, the incidence rate of sore throat, hoarseness, and dysphagia at the 24-hour follow-up had no difference between the group PPT and group TT.

In conclusion, the improved prone position ventilation tube shows a promising role for reducing the incidence of tube displacement.

## References

[1] Fineman LD, LaBrecque MA, Shih MC, Curley MAQ (2006). Prone positioning can be safely performed in critically ill infants and children. Pediatric Critical Care Medicine.

[2] Harris PD, Farmery AD, Patel CK (2009). The challenges of positioning an infant undergoing optical coherence tomography under general anesthesia. Paediatric anaesthesia.

[3] Soundararajan N, Cunliffe M (2007). Anaesthesia for spinal surgery in children. British journal of anaesthesia.

[4] Chui J, Craen RA (2016). An update on the prone position: Continuing Professional Development. Canadian journal of anaesthesia=Journal canadien d’anesthesie.

[5] Zou W, Zhang W, Li X, Guo Q (2013). A randomized crossover comparison of the prone ventilation endotracheal tube versus the traditional endotracheal tube in pediatric patients undergoing prone position surgery. Paediatric anaesthesia.

[6] Edgcombe H, Carter K, Yarrow S (2008). Anaesthesia in the prone position. British journal of anaesthesia.

[7] Santhosh MC (2013). Comparison of tube-taping versus a tube-holding device for securing endotracheal tubes in adults undergoing surgery in prone position. Acta anaesthesiologica Belgica.

[8] Singh G, Manikandan S, Neema PK (2011). Endotracheal tube fixation in neurosurgical procedures operated in prone position. Journal of anaesthesiology, clinical pharmacology.

[9] Buckley JC, Brown AP, Shin JS, Rogers KM, Hoftman NN (2016). A Comparison of the Haider Tube-Guard(R) Endotracheal Tube Holder Versus Adhesive Tape to Determine if This Novel Device Can Reduce Endotracheal Tube Movement and Prevent Unplanned Extubation. Anesth Analg.

